# Incidence of and Risk Factors for Mastoiditis after Intensity Modulated Radiotherapy in Nasopharyngeal Carcinoma

**DOI:** 10.1371/journal.pone.0131284

**Published:** 2015-06-26

**Authors:** Ji-Jin Yao, Guan-Qun Zhou, Xiao-Li Yu, Ling-Long Tang, Lei Chen, Yan-Ping Mao, Li Lin, Lu-Lu Zhang, Jian-Yong Shao, Ying Guo, Jun Ma, Ying Sun

**Affiliations:** 1 Department of Radiation Oncology, Sun Yat-sen University Cancer Center, State Key Laboratory of Oncology in South China, Collaborative Innovation Center for Cancer Medicine, Guangzhou, 510060, Guangdong Province, People’s Republic of China; 2 Department of Radiation Oncology, Sun Yat-sen Memorial Hospital, Sun Yat-sen University, Guangzhou, People’s Republic of China; 3 Department of Pathology, Sun Yat-sen University Cancer Center, State Key Laboratory of Oncology in South China, Collaborative Innovation Center for Cancer Medicine, Guangzhou, 510060, Guangdong Province, People’s Republic of China; 4 Department of Medical Statistics and Epidemiology, State Key Laboratory of Oncology in South China, Sun Yat-sen University Cancer Center, Guangzhou, People’s Republic of China; Chang-Gung University, TAIWAN

## Abstract

**Purpose:**

To report the incidence of and risk factors for mastoiditis after intensity-modulated radiotherapy (IMRT) in nasopharyngeal carcinoma (NPC).

**Patients and Methods:**

Retrospective analysis of pretreatment and follow-up magnetic resonance imaging (MRI) data for 451 patients with NPC treated with IMRT at a single institution. The diagnosis of mastoiditis was based on MRI; otomastoid opacification was rated as Grade 0 (none), 1 (mild), 2 (moderate) or 3 (severe) by radiologists blinded to clinical outcome. This study mainly focused on severe mastoiditis; patients were divided into three groups: the G0M (Grade 0 mastoiditis before treatment) group, G1-2M (Grade 1 to 2 mastoiditis before treatment) group and G3M (Grade 3 mastoiditis before treatment) group. The software SAS9.3 program was used to analyze the data.

**Results:**

For the entire cohort, the incidence of Grade 3 mastoiditis was 20% before treatment and 31%, 19% and 17% at 3, 12 and 24 months after radiotherapy, respectively. In the G0M group, the incidence of severe mastoiditis was 0% before treatment and 23%, 15% and 13% at 3, 12 and 24 months after radiotherapy, respectively. Multivariate analysis revealed T category (*OR*=0.68, 95% *CI* = 0.469 to 0.984), time (*OR*=0.668, 95% *CI* = 0.59 to 0.757) and chemotherapy (*OR*=0.598, 95% *CI* = 0.343 to 0.934) were independent factors associated with severe mastoiditis in the G0M group after treatment.

**Conclusions:**

Mastoiditis, as diagnosed by MRI, occurs as a progressive process that regresses and resolves over time in patients with NPC treated using IMRT.

## Introduction

Nasopharyngeal carcinoma (NPC) has an extremely unbalanced endemic distribution and is most prevalent in southern China where the incidence is between 15 and 50 per 100,000 [1]. Mastoiditis, mastoid with effusion, is relatively common both before and after radical radiotherapy in patients with NPC. The reported rates of mastoiditis following irradiation range from 15 to 50% [2–4]. Mastoiditis may cause significant health problems [5], such as a sensation of fullness or pressure in the ear, tinnitus, pain, otorrhea and even hearing loss. However, protection of the mastoid is difficult, particularly in patients with T3/T4 disease in whom the Eustachian canal (EC) or the surrounding structures (tensor veli palatini muscle, cartilage, nerves) may be extensively invaded or involved, which can result in the development of negative pressure in the middle ear (ME) and transudation of serous fluid into the mastoid [6].

The introduction of intensity-modulated radiotherapy (IMRT) has greatly improved the ability to distribute radiation doses more precisely [7, 8]. Compared to two-dimensional conventional radiotherapy (2D-CRT), IMRT can reduce the volume of the area of the mastoid receiving a high dose and decrease the risk of toxicity [8]. However, previous research on mastoiditis has mostly been based on patients treated with 2D-CRT, and data on the incidence and tumor-related factors associated with mastoiditis after IMRT are lacking. Therefore, we conducted a retrospective study to investigate the incidence of severe mastoiditis at different intervals after IMRT and to identify risk factors associated with mastoiditis after IMRT. The objective of this study was to provide a better understanding of the effect of radiation on severe mastoiditis in patients with NPC treated using IMRT.

## Materials and Methods

### Study population

Approval for retrospective analysis of the patient data was obtained from the ethics committee of Sun Yat-sen University Cancer Center. The Ethics committee of Sun Yat-sen University Cancer Center also waived the need for written consent because this was a retrospective study; verbal consent was obtained from the patients via telephone and documented in the informed consent form if the patient agreed to participate in this study. Between January 2009 and April 2010, 498 patients with newly-diagnosed, non-metastatic, histologically-proven NPC were treated with IMRT at our center. Of these 498 patients, 47 were excluded due to incomplete MRI information; this study was based on the remaining 451 patients. The Supplementary S1 file was a case-based collection of personal informaton including gender, age, pathotogy, treatment, T catetgory, N category and the grade of mastoiditis.

### Clinical staging

All patients underwent pre-treatment evaluations (complete medical history, physical examination, hematology and biochemistry profiles, MRI of neck and nasopharynx, chest radiography, abdominal ultrasonography, and whole body bone scan. Positron emission tomography (PET-CT) was performed on 133 (29.5%) patients. All patients were restaged according to the 7th edition of the International Union against Cancer/American Joint Committee on Cancer (UICC/AJCC) staging system [9]; all MRI data and clinical records were separately reviewed by two radiologists specializing in head and neck cancer to minimize heterogeneity in restaging.

### Treatment

All patients received IMRT to treat the primary tumor and upper neck area above the caudal edge of the cricoid cartilage. Total doses of 68–70 Gy in 30–33 fractions at 2.13–2.27 Gy/fraction to the planning target volume (PTV) of GTV-P, 60 Gy to the PTV of CTV-1 (high-risk regions), 54 Gy to the PTV of CTV-2 (low-risk regions and neck nodal regions), and 60–68 Gy to the nodal gross tumor volume (GTV-N) in 30–33 fractions were prescribed. Treatment was delivered once daily with five fractions per week.

During the time of the study, institutional guidelines recommended no chemotherapy for stage I to IIA, concurrent chemoradiotherapy for stage IIB, and concurrent chemoradiotherapy +/- neoadjuvant/adjuvant chemotherapy for stage III to IVA-B, as defined by the 6th edition of the UICC/AJCC staging system for NPC [10]. Overall, 66 (14.6%) patients were treated with IMRT alone and 385 (85.4%) patients received chemotherapy; 50 patients with stage III/IVA-B disease received only IMRT alone. Reasons for deviation from the guidelines included advanced age, organ dysfunction or allergic reactions indicative of intolerance to chemotherapy. Neoadjuvant and adjuvant chemotherapy were cisplatin with 5-fluorouracil and/or docetaxel every three weeks for two or three cycles. Concurrent chemotherapy was cisplatin weekly or on days 1, 22 and 43 of IMRT.

### MRI

The region from the suprasellar cistern to the inferior margin of the sternal end of the clavicle was examined in all patients (supine position) before treatment and 3, 12 and 24 months after radiotherapy on a 1.5-Tesla MRI system (Signa CV/I; General Electric Healthcare, Chalfont St. Giles, United Kingdom) using a head-and-neck combined coil. T1-weighted fast spin-echo axial, coronal and sagittal images (repetition time, 500–600 ms; echo time, 10–20 ms) and T2-weighted fast spin-echo axial images (repetition time, 4,000–6,000 ms; echo time, 95–110 ms) were obtained then 0.1 mmol/kg body weight gadopentetate dimeglumine (Gd-DTPA; Magnevist; Bayer-Schering, Berlin, Germany) was intravenously injected and axial and sagittal spin-echo T1-weighted and coronal spin-echo T1-weighted fat-suppressed sequences were obtained. Diffusion-weighted magnetic resonance imaging (DWI) using line scan diffusion images was also performed with a pelvic phased-array coil (*b* values of 5 and 800 s/mm^2^). Section thickness was 5 mm (1 mm interslice gap) for the axial plane and 6 mm (1 mm interslice gap) for the coronal and sagittal planes.

### Image assessment and volume measurement

Image evaluation was performed separately by two radiologists and a clinician, each with more than 10 years’ experience in head and neck cancer. In cases with differential findings, the final decision was reached by consensus. The diagnose of mastoiditis was according to the criteria reported by Platzek et al. [11]as follows: a) fluid accumulation, increased contrast enhancement of the mastoid and restricted diffusion in the mastoid, which were defined as signs of mastoiditis; b) subperiosteal fluid collection, extracranial contrast enhancement adjacent to the mastoid and restricted extracranial diffusion adjacent to the mastoid, which were interpreted as signs of a subperiosteal abscess; and c) all subperiosteal abscesses were defined as severe mastoiditis, regardless of the volume of the opacified structure [12].

Due to a discrepancy of mastoid opacification ratio between the left and right mastoid in NPC patients, both sides were examined independently for this study. The pretreatment MRI and the MRIs taken 3, 12 and 24 months after radiotherapy completion were examined for each study subject. The MRI scanning images were conveyed to Advantage Workstation 4.4 (General Electric Company, USA). T2-weighted axial images were selected and the outline of the mastoid was traced on each axial image manually. Mastoid volume was calculated by volume rendering. Two observers (one radiologist and one other clinician), trained for using the software and delineating the mastoid and the mastoid opacification, contoured each mastoid opacification ratio independently. The average of the measurements was taken as the final volume.

**Fig 1:**
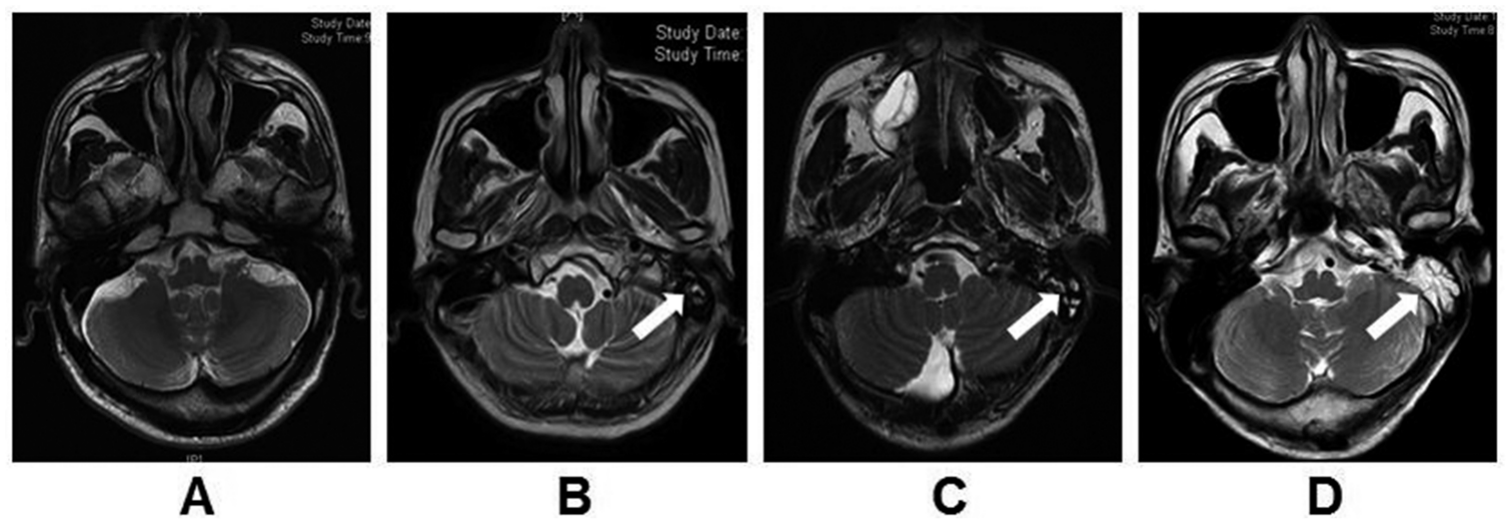
Representative T2 weighted MR images of different grades of mastoiditis. (A) Grade 0 mastoiditis, < 5% of the mastoid air cell is opacified; (B) Grade 1 mastoiditis, 6–33% of the mastoid air cell is opacified; (C) Grade 2 mastoiditis, 34–67% of the mastoid air cell is opacified; (D) Grade 3 mastoiditis, 68–100% of the mastoid air cell is opacified. A high-signal intensity area (white arrow) is observed in mastoid air cells in the left ear; the mastoid air cells in the right ear appear normal.

Mastoid opacification was used as a radiologic surrogate for mastoid effusion and was scored as Grade 0 (< 5% of the volume of the opacified structure), 1 (6–33%), 2 (34–67%) or 3 (68–100%) [6], as illustrated in Fig 1. Severe (Grade 3) mastoiditis was used as the end point of the study; most patients with mild or moderate mastoiditis have no clinical symptoms and the majority of symptoms in the ear (including tinnitus, pain, otorrhea and hearing loss) are due to severe mastoiditis [13].

### Patient follow-up and statistical analysis

Complete pretreatment and follow-up MRI data was available for all patients. Patients were followed up at least every 3 months in the first 3 years and every 6 months thereafter. Routine follow-up care included a complete head and neck examination, hematology and biochemistry profiles, chest radiography and abdominal sonography. Follow-up MRI of the neck and/or nasopharynx was performed every 6–12 months, especially for cases with suspected tumor recurrence or radiotherapy-induced complications. As the timing of the medical examinations after treatment was not exactly consistent, mastoiditis was evaluated at 3, 12 and 24 months after radiotherapy in this study.

All analyses were performed with SAS9.3 software (SAS, Inc; Cary, NC, USA). A cut-off point at 50 years old was used to separate the age of patients. The potential risk parameters, such as age, gender, World Health Organization (WHO) histological grade, T classification, N classification and use of chemotherapy, were all categorical variables, described with measures of absolute number and percentage (%). Then a generalized linear mixed model (GLMM) was applied to identify the risk factors from clinicopathological characteristics. The chi-square test was used to compare ratios between the groups. *P*-value < 0.05 was considered statistically significant.

## Results

### Subclassification of patients

The severity of mastoiditis before radiotherapy may have confounding effects on the occurrence of post-radiotheapy Grade 3 mastoiditis, therefore patients in the current study were divided into three groups: the G0M group who had Grade 0 mastoiditis before treatment, the G1-2M group who had Grade 1 or 2 mastoiditis before treatment and the G3M group who had Grade 3 mastoiditis before treatment. In the G3M group, the male: female ratio was 3.1:1 (134 males, 43 females), the median age was 44.7 years (range, 11–78 years), 164/177 (92.7%) of patients had World Health Organization (WHO) type 2 disease and 13/177 (7.3%) had WHO type 1 disease, and 97/177 (54.8%) had N0/N1 disease and 80/177 (45.2%) had N2/N3 disease. The gender, age, histology and N category distributions of the G0M group were not significantly different to the G3M group (all *P* > 0.05). However, the frequency of T1/T2 disease in the G0M group was 38.5% (258/670) compared to 10.2% (18/177) in the G3M group (P < 0.001). Patient characteristics were shown in Table 1.

**Table 1:** Clinicopathological features of the 451 patients with NPC treated using IMRT.

Characteristic	No.	%
Age, years		
<50	324	71.8
≥50	127	28.2
Gender		
Male	332	73.6
Female	119	26.4
Pathologic features		
WHO Type 1	25	5.5
WHO Type 2	426	94.5
T category*		
T1	47	10.4
T2	96	21.3
T3	196	43.5
T4	112	24.8
N category*		
N0	68	15.1
N1	170	37.7
N2	173	38.3
N3	40	8.9
Stage group*		
I	16	3.5
II	68	15.1
III	222	49.2
IVA-B	145	32.2
Chemotherapy		
Yes	385	85.4
No	66	14.6
Mastoiditis grades		
Grade 0	670	74.3
Grade 1	34	3.8
Grade 2	21	2.3
Grade 3	177	19.6

IMRT, intensity modulated radiotherapy; WHO, World Health Organization.

*According to the American Joint Committee on Cancer, 7^th^ edition.

### Incidence of mastoiditis

**Fig 2:**
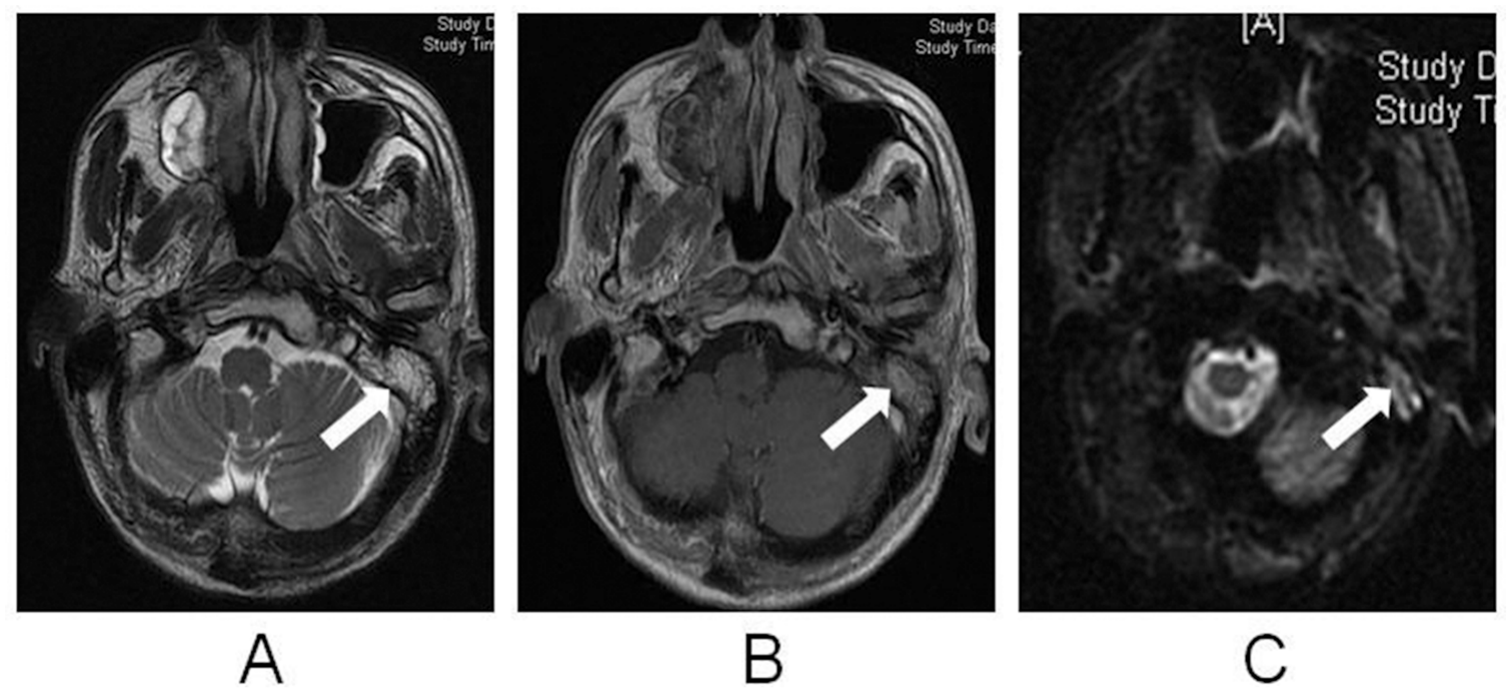
MRI images of a 45-year-old male patient with nasopharyngeal cancer and mastoiditis in the left ear. (a) Axial T2W image; (b) T1W contrast-enhanced image with fat saturation; (c) DWI (*b* = 1000). Fluid accumulation with peripheral contrast enhancement and restricted diffusion is observed in the left mastoid (white arrow); the right mastoid appears normal.

A total of 451 patients with NPC (902 mastoid/ME complexes) were included in the final analysis. Based on the diagnostic criteria previously reported by Platzek et al. (11) (Fig 2), mastoiditis was confirmed in 232 (25.7%) of 902 mastoids. For the entire cohort, the incidence of Grade 3 mastoiditis was 20% before treatment and 31%, 19% and 17% at 3, 12 and 24 months after radiotherapy, respectively. Based on the presence or absence of severe mastoiditis before treatment, the mastoid/ME complexes were divided into three groups: the G0M group containing 670/902 (74.3%) mastoid/ME complexes, the G1-2M group containing 55/902 (6.1%) mastoid/ME complexes and the G3M group containing 177/902 (19.6%) mastoid/ME complexes. Based on the reference standard, the incidence of subperiosteal abscesses was 7% (12/177) in the G3M group at pretreatment.

**Fig 3:**
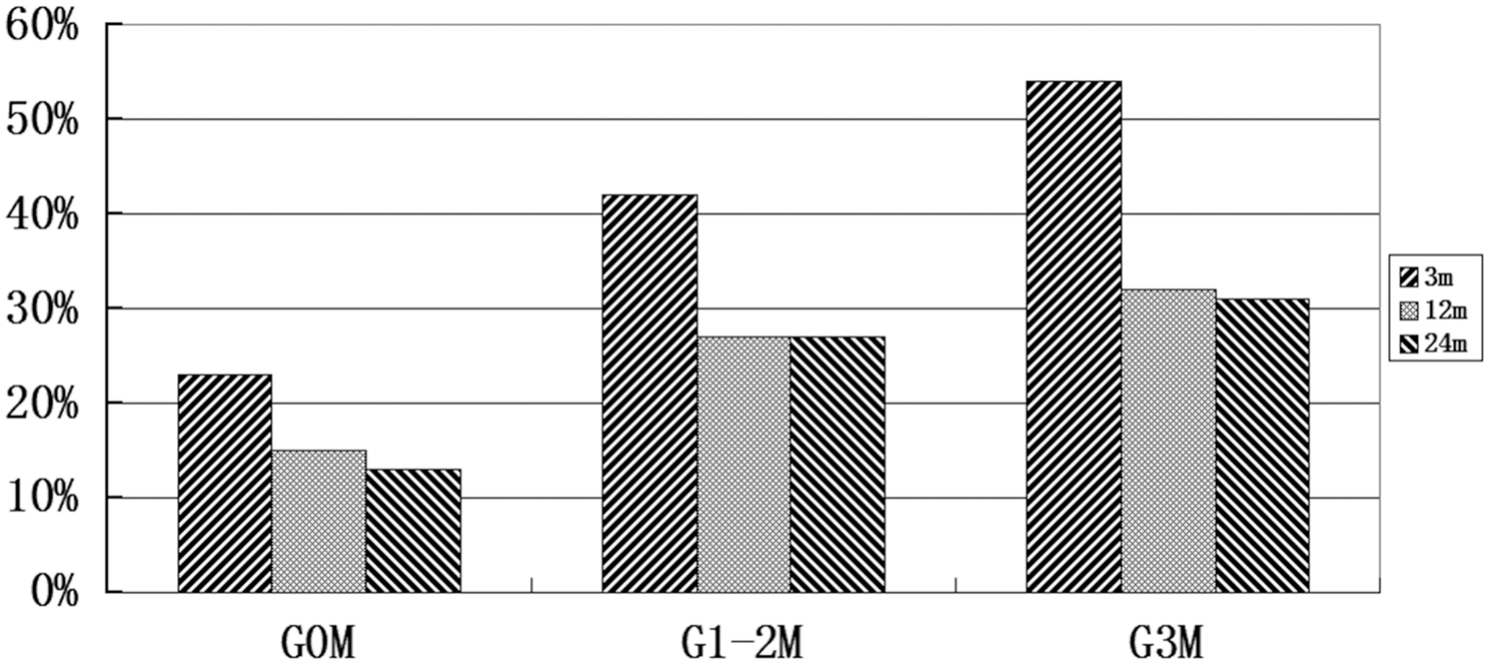
Incidence of severe (Grade 3) mastoiditis diagnosed by MRI at different follow-up times after intensity-modulated radiotherapy in patients with NPC stratified by the presence or absence of Grade 3 mastoiditis before treatment. The incidence of severe mastoiditis at 3, 12 and 24 months after radiotherapy was significantly higher in the G1-2M group (42%, 27% and 27%) than the G0M group (23%, 15% and 13%, respectively; all P<0.05).

The rates of severe mastoiditis by group, classification and follow-up interval are presented in Fig 3. The incidence of severe mastoiditis at 3, 12 and 24 months after radiotherapy was significantly higher in the G1-2M group (42%, 27% and 27%) than the G0M group (23%, 15% and 13%, respectively; all *P*<0.05). In the G3M group, the incidence of severe mastoiditis was 100% before treatment and 54%, 32% and 31% at 3, 12 and 24 months after radiotherapy. Overall, the incidence of mastoiditis diagnosed by MRI increased significantly in the first 3 months after radiation, and then reduced and remained similar between 1 and 2 years after radiotherapy.

### Risk factors for mastoiditis after IMRT

Multivariate analysis was performed to investigate the independent significance of various clinicopathological features as risk factors for mastoiditis after treatment in both groups (Table 2). The following parameters were included as variables: age (< 50 vs. ≥50 years), gender (male vs. female), histology (WHO type 1 vs. WHO type 2), T category (T1-2 vs. T3-4), N category (N0-1 vs. N2-3) and chemotherapy (no vs. yes). After forward inclusion of significant explanatory variables, T (tumor) category (*OR* = 0.68, 95% *CI* = 0.469 to 0.984), time (*OR* = 0.668, 95% *CI* = 0.59 to 0.757) and chemotherapy (*OR* = 0.598, 95% *CI* = 0.343 to 0.934) were found to be independent predictors of mastoiditis after treatment in the G0M group. Age, gender, histological category and N (node) category did not have any statistically significant association with mastoiditis after treatment in the G0M group.

**Table 2:** Multivariate analysis of predictors for the occurrence of post-radiotherapy Grade 3 mastoiditis.

	G0M group		G1-2M group		G3M group	
Variables	OR^†^	95%CI^†^	OR^†^	95%CI^†^	OR^†^	95%CI^†^
Histology	1.024	0.486–2.157			0.954	0.360–2.526
Treatment^‡^	0.598	0.343–0.934			1.800	0.667–4.861
Age^‡^	0.844	0.589–1.211	0.699	0.253–1.931	0.404	0.237–0.689
Gender	0.784	0.554–1.109	1.809	0.456–7.180	0.585	0.326–1.049
T^‡^	0.68	0.469–0.984	0.864	0.233–3.205	0.486	0.188–1.257
N	0.933	0.670–1.299	0.619	0.223–1.723	0.859	0.513–1.437
Time^‡^	0.668	0.590–0.757	0.706	0.489–1.019	0.595	0.487–0.725

*OR*, odds ratio; *CI*, confidences interval; G0M, grade 0 mastoiditis; G1-2M, grade 1–2 mastoiditis; G3M, grade 3 mastoiditis.

^†^ *OR* and 95%*CI* were calculated using a GLMM (generalized linear mixed model).

^‡^ Treatment, T category and time were significant risk factors of post-radiotherapy G3M in the G0M group. Age and time were found to be independent predictors for post-radiotherapy G3M in the G3M group.

**Note:** Due to the limited number of patients, as well as highly unbalanced classification of treatment and pathology in the G1-2M group, we couldn't get any result in multivariate analysis by GLMM.

In the G3M group, there was no significant association between gender, histology, T category, N category or chemotherapy and mastoiditis after treatment in the G3M group (*OR* = 0.585, 95% *CI* = 0.326 to 1.049; *OR* = 0.954, 95% *CI* = 0.36 to 2.526; *OR* = 0.486, 95% *CI* = 0.188 to 1.257; *OR* = 0.859, 95% *CI* = 0.513 to 1.437 and *OR* = 1.800, 95% *CI* = 0.667 to 4.861, respectively); however, age (*OR* = 0.404, 95% *CI* = 0.237 to 0.689) and time (*OR* = 0.595, 95% *CI* = 0.487 to 0.725; Table 2) were significant independent risk factors for mastoiditis after treatment in the G3M group. Due to the limited number of patients, as well as highly unbalanced classification of treatment and pathology in the G1-2M group, we couldn't get any result in multivariate analysis by GLMM.

### Subgroup analysis of the influence of T category, chemotherapy and age on mastoiditis after IMRT

Multivariate analysis revealed a significantly higher risk of mastoiditis after treatment for patients with T3/T4 disease in the G0M group; the incidence of mastoiditis at 3, 12 and 24 months after radiotherapy was 17%, 11% and 10% for patients with T1/T2 disease compared to 27%, 17% and 14% for patients with T3/T4 disease, respectively (*OR* = 0.68, 95% *CI* = 0.469 to 0.984). However, a similar trend was not evident in the G1-2M and G3M group (*OR* = 0.864, 95% *CI* = 0.233 to 3.205 and *OR* = 0.486, 95% *CI* = 0.188 to 1.257, respectively).

The effect of chemotherapy on the incidence of mastoiditis was also evaluated. In the G0M group, the incidence of mastoiditis in patients treated with radiotherapy alone (14%, 11% and 6% at 3, 12 and 24 months) was significantly lower than the incidence of mastoiditis (25%, 16% and 14% at 3, 12 and 24 months) for patients treated with cisplatin-based chemoradiotherapy (*OR* = 0.598,95% *CI* = 0.343 to0.934). However, there was no statistically significant difference in the incidence of mastoiditis after radiotherapy alone or cisplatin-based chemotherapy in the G3M group (*OR* = 1.800, 95% *CI* = 0.667 to4.861).

The effect of age on the incidence of mastoiditis was also evaluated. In the G3M group, the incidence of mastoiditis in patients with age ≥50 (64%, 46% and 46% at 3, 12 and 24 months) was significantly higher than the incidence of mastoiditis (48%, 25% and 23% at 3, 12 and 24 months) for patients with age<50 (*OR* = 0.404,95% *CI* = 0.237 to 0.689). However, a similar trend was not evident in the G0M and G1-2M group (*OR* = 0.844, 95% *CI* = 0.589 to 1.211 and *OR* = 0.699, 95% *CI* = 0.253 to 1.931, respectively).

## Discussion

IMRT has been widely accepted as an advanced radiation technique for the management of NPC [14–16]. However, data on the incidence of mastoiditis diagnosed by MRI in patients treated with IMRT is lacking. Therefore, it is reasonable to investigate the incidence of mastoiditis and risk factors on severe mastoiditis for NPC in the new era of IMRT. This study demonstrates that mastoiditis are associated with age, chemotherapy and T stages, and occurs as a progressive process that regresses and resolves over time in NPC patients treated with IMRT.

### Advantages of MRI for the diagnosis of mastoiditis

Past research mainly relied on single diffusion-weighted MRI for the diagnosis of mastoiditis [2, 12, 17–18]. For example, Nishimura et al. [2] and Polat et al. [12] only evaluated the role of T2-weighted (T2W) images, while DWI or contrast-enhanced MRI was not evaluated. T2W images have a high sensitivity for effusion and inflammatory disease of the middle ear and mastoid air cells [2, 11]. However, based on our clinical experience, increased fluid signal intensity in the mastoid should not be interpreted as a sign of mastoiditis, especially if signs of mastoiditis are absent from other multi-parametric imaging. Thus, the use of single diffusion-weighted MRI alone may lead to false-positive diagnoses of mastoiditis [18, 19].

The diagnostic method for mastoiditis was mainly based on T2W images in the past [2, 19–20], while the role of DWI or contrast-enhanced MRI was not mentioned. In the present study, we evaluated the roles of T2W and T1W images with contrast enhancement combined with DWI, which distinctly differed to that of earlier works. Our selection of this imaging protocol was influenced by Platzek et al [11], who demonstrated that multiparametric MRI had high sensitivity and diagnostic accuracy in mastoiditis. Therefore, the use of this unique approach provided a measure of confidence in our results.

### Incidence of severe mastoiditis

Radiation is generally considered to play a significant role in the development of mastoiditis. Using T2-weighted MRI in a series of 114 patients, Nishimura et al. [2] reported the incidence of radiation-induced mastoiditis was 18%, 13% and 8% within 6 months, 6–12 months and 12 months after 2D-CRT, respectively. In the present study, the incidence of severe mastoiditis was 23%, 15% and 13% at 3, 12 and 24 months after IMRT, respectively, which is higher than the rates reported by Nishimura. This inconsistency is probably due to the different diagnostic modalities utilized in this study. Mastoiditis can usually not be histologically-confirmed. Many previous studies used clinical symptoms (in combination with imaging examinations) as the major diagnostic criterion for mastoiditis [2, 6, 21]. However, the majority of cases of mastoiditis tend to be asymptomatic or have very vague symptoms, which leads to an omission bias and therefore an underestimation of the true incidence of mastoiditis. MRI has high accuracy for the identification of mastoiditis and concurrent subperiosteal abscesses [11] and was employed to diagnose mastoiditis in the current study.

In our study, the incidence of radiation mastoiditis was dependent on the status of mastoid at pretreatment. The incidence of radiation mastoiditis was quite high among our patients whose mastoids had mile to moderate mastoiditis at pretreatment. And we also noted patients had none mastoiditis before treatment present a relatively lower incidence of severe mastoiditis at each examination point. The reason may be mild to moderate mastoiditis were more likely to induce the occurrence of severe mastoiditis under radiation.

### Confounding risk factors for mastoiditis after IMRT

Gender, age, pathology, T category, N category and treatment with cisplatin were all examined as confounding factors in multivariate analysis. Due to the design of the study, the effect of time and other potential confounders on radiation-induced mastoiditis could be isolated. T category and cisplatin chemotherapy were independently associated with radiation-induced mastoiditis in the G0M group. Younger age (< 50 years) was a protective factor in the G3M group, consistent with the pathophysiology of this deficit [22]; however, we did not observe any association between age and radiation-induced mastoiditis in the G0M and G3M group.

Cisplatin ototoxicity is well documented [23,24], and its relationship with radiation-induced serous otitis media (SME) was described by Walker et al. [25]. In our study, we demonstrated a significant association between cisplatin chemotherapy and radiation-induced mastoiditis in the G0M group. Although the exact mechanisms associated with this observation remain unknown, cisplatin chemotherapy may increase the risk of mastoiditis if combined with radiation. However, this observation may be due to the limited number of patients, because we had only 10 patients (15 ears) received cisplatin chemotherapy in the G3M group.

### Incidence of mastoiditis after IMRT over time

Multivariate analysis indicated that time was independently associated with the development of mastoiditis after IMRT. The incidence of radiation-induced mastoiditis increased markedly at 3 months then reduced significantly at 3 to 12 months and reduced slightly 12 to 24 months after radiotherapy. Two potential factors may explain the increased incidence of mastoiditis shortly after IMRT: (1) the dose of radiation to the mastoid air cells and Eustachian canal (EC); and (2) short-term reactive edema in the EC after radiotherapy. Reactive edema may be gradually absorbed and removed, which would lead to a lower incidence of mastoiditis as time progresses.

Furthermore, the incidence of mastoiditis in the G3M group reduced significantly between pretreatment and 3 months after radiotherapy. Shrinkage of the tumor as a result of radiotherapy may remove pressure and restore the normal structure of the EC or radiation may directly kill the bacteria responsible for mastoiditis [26–28].

## Conclusions

To the best of our knowledge, this is the largest single-institutional study to investigate mastoiditis in NPC after IMRT. The risk of postradiation mastoiditis diagnosed by MRI increased rapidly at 3 months and then reduced at 1–2 years after IMRT. Radiotherapy-induced mastoiditis in NPC is a progressive process that regresses or resolves over time.

## Supporting Information

S1 File Case-based collection of personal informaton including gender, age, pathotogy, treatment, T catetgory, N category and the grade of mastoiditis for each patient. (XLS)
